# Optimization and Thermoeconomics Research of a Large Reclaimed Water Source Heat Pump System

**DOI:** 10.1155/2013/893020

**Published:** 2013-09-05

**Authors:** Zi-ping Zhang, Fang-hui Du

**Affiliations:** ^1^Department of Air Conditioning and Refrigeration Engineering, Hebei University of Engineering, Handan 056038, China; ^2^Hebei Sanyou Energy Technology Development Co., Ltd., Shijiazhuang 050021, China

## Abstract

This work describes a large reclaimed water source heat pump system (RWSHPS) and elaborates on the composition of the system and its design principles. According to the characteristics of the reclaimed water and taking into account the initial investment, the project is divided into two stages: the first stage adopts distributed heat pump heating system and the second adopts the combination of centralized and decentralized systems. We analyze the heating capacity of the RWSHPS, when the phase II project is completed, the system can provide hydronic heating water with the supply and return water temperature of 55°C/15°C and meet the hydronic heating demand of 8 million square meters of residential buildings. We make a thermal economics analysis by using Thermal Economics theory on RWSHPS and gas boiler system, it is known that the RWSHPS has more advantages, compared with the gas boiler heating system; both its thermal efficiency and economic efficiency are relatively high. It provides a reference for future applications of the RWSHPS.

## 1. Introduction 

Nowadays, as we known that the energy input for building operation is mainly constituted by fossil energy carriers, therefore building operation is responsible for a large share in global greenhouse gas emissions [1]. As the development of urban construction, accelerates heating gap exists in many cities in china. With the energy shortage and the requirement of having a better environment and in order to decrease the energy demand and the related emission, it is necessary for us to develop and use renewable energy source in the urban heating. Rosen [2] describes the methods of dealing with global warming through the development and utilization of nonfossil energy. Therefore, to improve the efficiency of energy conversion, the reasonable use of renewable energy has become an important research direction. 

Reclaimed water, which is the sewage plant secondary or tertiary effluent in this paper, has the advantages of the concentrated water flow, high temperature (compared to surface water), being relatively clean, and so forth, so it is the ideal heat source for heat pump units. The wastewater treatment plant is generally located in the edge of the city and in recent years; the reality is that urban construction has been extended within closer range from the wastewater treatment plant, and the high cost of laying in the water distribution network will no longer be the main problem of no large-scale application in water source heat pump works. Taking the Reclaimed water as the heat source of the urban hydronic heating can achieve efficient recycling of urban sewage heat energy, turning waste into treasure, which fully embodies the concept of the development of recycling economy, greatly improves the energy efficiency of the city, and reduces the city's dependence on the fossil fuel. 

The project uses the city's Reclaimed water, which is the sewage plant secondary or tertiary effluent and contains a large number of renewable energy, to develop the new energy technologies and to complement the traditional district heating gap. The RWSHPS use the heat pump technology to extract the low grade energy of the renewable water, and then provided them to many kinds of buildings nearby such as residential areas, schools and industrial park for heating and cooling. 

The sewage plant covers an area of 540 acres with the designed treatment capacity of 700,000 tons and nowadays the daily output reaches 500,000 tons of Reclaimed water; it is currently one of the largest sewage treatment plant in china. The waste water generally comes from urban sewage and industrial wastewater in the east of Beijing-Guangzhou line. The temperature of the effluent is 22 ~ 27°C in summer and 14 ~ 19°C in winter. The average effluent available flow is 25000 t/h, and the available temperature lift or drop is about 10°C. The hot and cold medium for heating and cooling is the effluent of the wastewater treatment plant; it will be delivered to each consumer through the distribution network laying in project of phase I. Intake point of water catchment is located on the tertiary treatment outlet pipe which will extend to the Minxin River, so the water supply has high quality with no secondary pollution. Now the quality of tertiary effluent water reaches level 1-A of sewage treatment plant emissions standards; it is close to clean water. Water hardness and metal salt content are relatively small; the PH value is 6–9, relatively moderate. So the corrosion of the Reclaimed water to the heat pump unit is very small. It can be directly taken into the unit heat exchanger.

**Figure 1:**
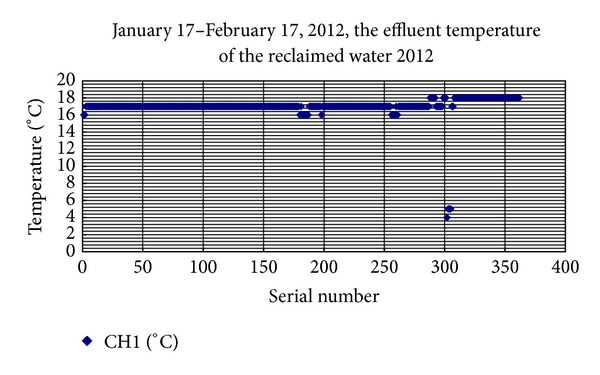
Water outlet temperature in the winter of 2012, January 17–February 17.

As shown in Figure 1, the average water temperature of the water effluent of the coldest month in the winter was 17-18°C, using Hong Chang HC2102R, paperless recorder with pt100 thermal resistance probe continuous data collection.

## 2. System Design Options

### 2.1. The Project Phase I Uses the Distributed Heat Pump Heating System

**Figure 2:**
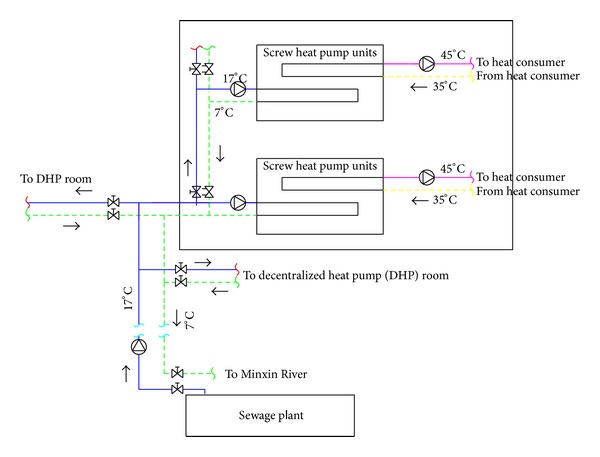
Schematic diagram of phase I project of heat pump system.

According to the New User's heating demand, the heating area is 2.02 million square meters, subdivided into 8 blocks; each block has a heat pump equipment room. According to the actual situation of the communities construction, heating demand is not consistent, even if it is in the same community; the end user's load rate also varied, taking into account the projects initial investment and rational use of water resources; the project phase I takes the form of the distributed heat pump heating system; all adopt the screw heat pump units; its exergy loss coefficient decreases with decreasing loading rate [3]; it is suitable for variable load state. In the project phase I, the 17°C Reclaimed water directly provided by the competent network is sent to the local heat pump equipment room as the heat source of the heat pump, and then users are provided with hot water of 45/35°C. The system diagram is shown in Figure 2.

Adopting the distributed heat pump heating system can realize large-scale district heating/cooling and also has dispersed flexibility. It not only improved the efficiency of the use of renewable energy but also filled the gap of local heating. Centralized return water can be used in the village and the city's green irrigation, which will save water resources and create additional profit for the enterprises.

### 2.2. The Second Phase of the Project Adopts Two-Stage Series Heat Pump Units

 Currently, the conventional single-stage heat pump [4] can only provide hot water of about 50°C but is also limited by the temperature of heat-source water; when the heat source water temperature is reduced, the pressure ratio of the compressor is increased, the gas transmission coefficient is reduced, so that the degree of which compression cycle process deviated from the isentropic process increases, the heat of cycle declines, the power consumption is increased, and the performance of the system decreases rapidly. Based on the theory of heat pump, some experts and scholars concluded a double-stage coupling heat pump heating system (DSCHP) which is suitable with characteristics of cold regions [5]. Two sets of single-stage heat pump system was integrated in the system by water circulation line and coupled together to form a suitable heat pump heating system for cold regions.

In order to meet the needs of 8 million square meters of building heating, the capacity of heat pump units is very big; however, the cost of conventional two-stage compression type high temperature heat pump unit with large capacity is higher; if we choose the multilevel compression high temperature water source heat pump units will lead to further increases in initial investment, so this paper uses the coupling system of the main heat pump station and local heat pump station. There are two-stage series heating units both in the main heating station and local heat pump room. The project phase II system can realize heating with large temperature difference at the same time reducing the initial investment.

**Figure 3:**
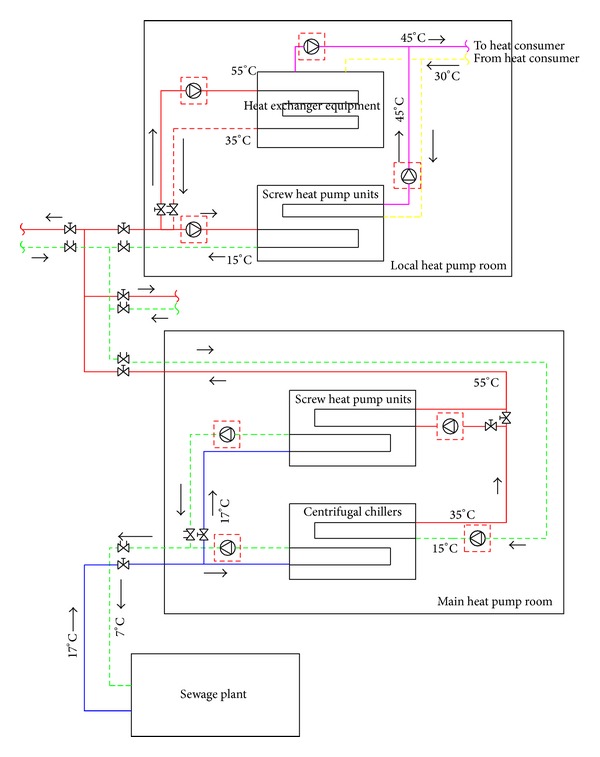
Schematic diagram of phase II project of heat pump system.

The project phase II system has three main components, that is, to the main pump station, a distributed power system, and user side heat exchange station. The second stage heat pump system schematic diagram is shown in Figure 3.

As shown in Figure 3, due to the community's construction scheduling in the second phase project, the 10°C temperature difference between supply water and return water mentioned before already cannot meet the heating requirements [6], so we will build the main heat pump plant in the second phase of the project, and it still uses the renovated water pipe network which is constructed in the first phase project to distribute the Reclaimed water. The second phase of the main heat pump plant is located 200 m to the north of the wastewater treatment plant and takes the form of concentration and indirect system. The sewage plant effluent will be led into large centrifugal pump units through 4 DN1200 steel pipe; the biggest water inflow is the biggest effluent of the wastewater treatment plant, about 20833 t/h. The main electric heat pump plant will be built in the Qiaodong sewage treatment plant on the north side, using the tertiary effluent of the sewage treatment plant for cooling in summer and heating in winter.

Under the condition of heating, the main heat pump plant adopts the mode of two-stage heating. In the first stage, with the heat absorbed from the Reclaimed water, the centrifugal pump units heats the return water in middle loop to 35°C from 15°C and then the water of 35°C goes into the second stage of screw heat pump units in series connection, exchanging heat with another branch of the Reclaimed water and the latter will be heated from 35°C to 55°C. The main heat pump station can achieve total temperature rise of 40°C. We only run the first stage centrifugal heat pump units at part load heating season.

In order to make full use of the large temperature difference of heat source which the main supply network provides, the decentralized local heat pump room also takes the form of two-stage exchanging heat, the 55°C hot water flow into the plate heat exchanger first and the release of heat quantity to the hot water of the user side. It will provide users with 45°C hot water. 35°C backwater of the plate heat exchanger series goes into the evaporator of the heat pump units and releases heat quantity for the second time; the temperature of medium water reduces from 35°C to 15°C. The temperature of water supply in the side of heat consumer is 45°C, return 30°C. 

## 3. Analysis and Discussion

### 3.1. The Heating Capacity of the Project

The sewage flow of the Qiaodong sewage treatment plant is about 600,000 tons per day; namely, the effluent can reach 25000 t/h. The effluent temperature is at an average of 17°C in winter, and it can reduce to 7°C after the release of heat. The diameter of the main supply and return water pipe in phase I of the project is DN1000, and the designing flow rate is 2.5 m/s; the heat pump units of the heating coefficient of COP_h_ take 4.0 provisionally supply/return water temperature of 17°C/7°C. As the new buildings are energy saving buildings, so we take the 40 W/m^2^as the average heating load index of Shijiazhuang region. The heating capacity is limited by the pipe network transmission capacity. Computation formula [7] is as follows:

(1) $$\begin{array}{c} Q=Q^{\prime }+W,\\ \text{COP}=\frac{Q}{W}=4.0,\\ Q^{\prime }=\frac{cM\Delta t}{3.6},\\ M=3600\pi R^{2}v\cdot \rho_{\text{water}}, \end{array}$$

where *c* is the water specific heat capacity, taking 4.187 kJ/(kg·°C); *M* is the mass flow, with the unit being kg/s, project phase I the main transport capacity; Δ*t* is the temperature difference between supply and return water; *Q*′ is the heat quantity extracted from the tertiary treatment effluent; *Q* is the total heat quantity to heat consumer; *W* is the input power of heat pump unit; *R* is the radius of the main supply and return water pipe and is 0.5 m; *v* is the designing flow rate of the Reclaimed water in the main pipe and is 2.5 m/s.

After calculation, it can be concluded that directly using the effluent water to extract 10°C temperature difference can meet heating of 2.7 million square meters, and it can cover the heating needs of project phase I of 2 million square meters.

Upon the completion of the second phase system, it can provide high temperature heating water, and the supply and backwater temperature is 55/15°C, with temperature lift of 40°C. If the flow rate is still 7065 t/h, taking into account the 1% of heat loss, it still can meet the heating area of 8.1 million square meters needed for 325 MW heat load. 

### 3.2. Thermoeconomics Modeling and Analysis of the Heating Systems

Taking the traditional analysis or evaluation method such as exergy analysis method and energy analysis method to evaluate energy conversion system, it just stood in the angle of the thermodynamics perfect degree of the system, without taking into account the cost of the economic factors along with the high efficiency; thus it is unable to comprehensively evaluate the advantages and disadvantages of the energy conversion systems.

Dincer [8] puts forward the concept of “exergy cost,” integrates the two methods of energy system evaluation, which are economic cost analysis and thermodynamic exergy analysis, and creates the concept of thermoeconomics in terms of energy system evaluation. Through combining the cash balance equations and the structure of the system, we will use the principle of grey box to establish the thermal economics model for the RWSHPS and gas boiler heating system.

#### 3.2.1. Thermoeconomics Modeling of Distributed Heat Pump Systems in Project Phase I

**Figure 4:**
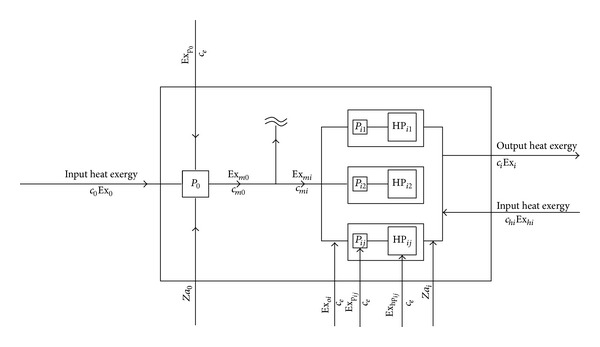
The thermal economics model of distributed heating pump system.

The entire system can be seen as a big gray box; there are three subsystems in the gray box, which contains the main circulating pump *P*_0_, distributed pump *P*_*ij*_, and local heat pump units HP_*ij*_; because the pipe network and indoor heating form of the user side are the same, so we do not consider the side of the user's part. The thermal economics model of project phase I is as shown in Figure 4.

The overall cash balance equations and the corresponding exergy balance equation of the project phase I are as follows:

(2) c0Ex0+ceExP0+Za0  +∑i{∑jceExPij+∑jceExHPij+chiExhi+ceExOi+Zai} =∑iciExi (i=1,2,…,8),

(3) Ex0+ExP0+∑i{∑jExPij+∑jExHPij+ExOi+Exhi}  −∑Exdest=∑iExi.

The heat source of the Reclaimed water source heat pump can be seen as the steady-state steady-flow process in the actual project, with the working fluid dynamic potential energy relative to the enthalpy being often negligible, so the specific enthalpy exergy of the water entering in the system is:

(4) $$\begin{array}{c} e_{x0}=\left(h-h_{0}\right)-T_{0}\left(s-s_{0}\right). \end{array}$$

Exergy and exergy efficiency of the system is the ratio of income and payment of exergy, so the calculation formula of exergy efficiency *ε*_Π_ of the distributed heat pump system is as follows:

(5) $$\begin{array}{c} \varepsilon_{\Pi }=\frac{\sum_{i}\text{E}{\text{x}}_{i}}{\text{E}{\text{x}}_{0}+\text{E}{\text{x}}_{P_{0}}+\sum_{i}\left(\text{E}{\text{x}}_{O_{i}}+\text{E}{\text{x}}_{hi}\right)+\sum_{i,j}\left(\text{E}{\text{x}}_{P_{ij}}+\text{E}{\text{x}}_{{\text{HP}}_{ij}}\right)}, \end{array}$$

where *e*_*x*0_ is the specific enthalpy exergy of the Reclaimed water entered into the system, kJ/kg and H is the specific enthalpy of Reclaimed water. When Reclaimed water temperature is at 17°C, its specific enthalpy is 71.32 kJ/kg; when it is 45°C, it is equal to 188.42 kJ/kg; the backwater 35°C, equal to146.59 kJ/kg. *h*_0_ is the specific enthalpy of the reference state, −0.05 kJ/kg; *T*_0_ is the temperature of the reference state and is 273 k; *s* is the specific entropy of Reclaimed water. When it is 17°C, its specific entropy is 0.2533 kJ/(kg·K); when it is 45°C, its entropy is 0.6386 kJ/(kg·K); and when it is 35°C, its entropy is 0.505 kJ/(kg·K). *s*_0_ is the specific entropy of the reference state, −0.0002 kJ/(kg·K); HP_*ij*_ is the heat pump unit, that is, the heat pump unit *j* in the *i*th local heat pump room; Ex_0_ is the heat exergy input to the system along with the Reclaimed water, kW; *C*_0_ is the unit heat exergy cost of input, yuan/kW.

Taking the known data above into (3), (4), and (5), and we can get that the exergy efficiency of the distributed heat pump heating system in project phase I is 66.2%.

#### 3.2.2. Thermoeconomics Analysis of Heat Pump System in Project Phase II

**Figure 5:**
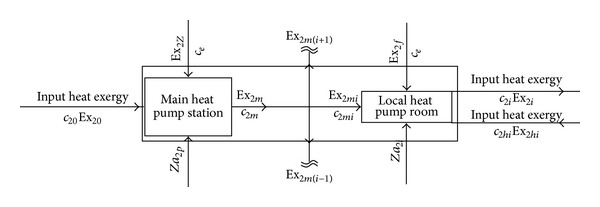
The thermoeconomics model of phase II project system.

The heating demand ultimately will reach 8 million square meters; because all user's heating load is not sure now, we suppose that the entire heating area (planning) is divided into 27 cells, each of 300,000 square meters. We have established the thermoeconomics analysis model for project phase II, as shown in Figure 5. 

The cash balance equation and the corresponding exergy balance equation of project phase II are

(6) c20Ex20+ceEx2Z+Za2p +27(ceEx2f+c2hiEx2hi+Za2i)=27c2iEx2i,Ex20+Ex2Z+27(Ex2f+Ex2hi)−∑Ex2dest=27Ex2i.

The calculation formula of exergy efficiency *ε*_2Π_ of the project phase II system is as follows:

(7) $$\begin{array}{c} \varepsilon_{2\Pi }=\frac{27\text{E}{\text{x}}_{2i}}{\text{E}{\text{x}}_{20}+\text{E}{\text{x}}_{2Z}+27\left(\text{E}{\text{x}}_{2f}+\text{E}{\text{x}}_{2hi}\right)}\times 100\%. \end{array}$$

After calculation, we can get that *ε*_2Π_ is 60.6%. 

#### 3.2.3. Thermoeconomics Model of Gas Boiler Heating System

The fuel of the gas boiler system is mainly natural gas. In the combustion process, natural gas and oxygen are mixed thoroughly, so the combustion is sufficient, containing a small amount of dust in the flue gas emissions and almost no nitrogen oxide and sulfide. Natural gas belongs to clean energy, so using gas boiler for heating is friendly to our environment. The Shijiazhuang government makes some policies and regulations [9] against atmospheric pollution and the environmental improvement, which pointed out that the city's distributed coal-fired boilers must be dismantle or transformed in the time of one year; central heating cannot meet the new district heating can be used in addition to the water source heat pump centralized heating can also choose other than gas-fired boiler system. The gas boiler system can be selected for the newly built residential area as a heating mode when urban centralized heat supply cannot meet the needs. 

**Figure 6:**
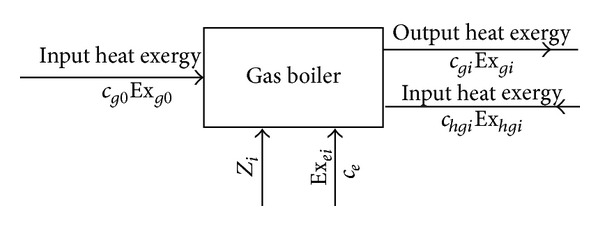
The thermoeconomics model of the gas boiler heating system.

In accordance with the principles of the black box, taking the gas boiler system in one district as a whole system, tracking the energy flow and cash flow of the system, the thermoeconomics model of the gas boiler heating system is shown in Figure 6, where *c*_*g*0_ is the unit heat exergy cost of input natural gas, yuan/kW; Ex_*g*0_ is the input heat exergy flow of the system, kW; Ex_*ei*_ is the gas boiler system (including boiler and boiler auxiliary equipment such as fans and pumps) input exergy flow electric, kW; *c*_*e*_ is the electric unit price, yuan/(kW·h); Z_*i*_ is the annual cost of the *i*th gas boiler heating room, yuan; Ex_*gi*_ is the output heat exergy flows of the gas-fired boiler system, kW; Ex_*hgi*_ is the heat exergy flows input into the gas boiler system along with the backwater, kW; *c*_*hgi*_ is the unit cost of the input heat exergy along with the backwater of the *i*th gas boiler room system, yuan/kW; *c*_*gi*_ is the unit cost of the output heat exergy of the gas boiler system, yuan/kW.

The cash balance equation and the corresponding exergy balance equation of the gas boiler system are

(8a) $$\begin{array}{c} c_{g0}\text{E}{\text{x}}_{g0}+c_{e}\text{E}{\text{x}}_{ei}+Z_{i}=c_{gi}\text{E}{\text{x}}_{gi}, \end{array}$$

(8b) $$\begin{array}{c} \text{E}{\text{x}}_{g0}+\text{E}{\text{x}}_{ei}+\text{E}{\text{x}}_{hgi}-\text{E}{\text{x}}_{g\text{dest}}=\text{E}{\text{x}}_{gi}. \end{array}$$

The heat load of 300,000 square meters of residential district is 12 MW; we can configure two 7 MW gas boiler [10] systems for district heating. The related formulas are as follows:

(9) $$\begin{array}{c} B_{1}=3.6\times 10^{6}\times \frac{Q_{1}}{\eta_{Ι}\cdot Q_{\text{ar}\cdot \text{net}}}\times 1.02,\\ \text{E}{\text{x}}_{g0}=\frac{B_{1}}{3600}\left(1-\frac{T_{g0}}{T_{g}}\right)\cdot Q_{\text{ar}\cdot \text{net}},\\ {\eta_{g}}_{\Pi }=\frac{\text{E}{\text{x}}_{gi}}{\text{E}{\text{x}}_{g0}+\text{E}{\text{x}}_{ei}}\times 100\%, \end{array}$$

where *B*_1_ is the gas consumption of the boiler system, m^3^/h; *η*_*Ι*_ is the thermal efficiency of the gas boiler, which is 88% when the capacity of the gas boiler is more than 7 MW; *Q*_ar·net_ is the low calorific value of the gas, 35530 kJ/m^3^; 1.02 is the additional coefficient of the hot water heat loss through the heat exchanger station; *T*_*g*0_ is the exhaust gas temperature, 298 K; *T*_*g*_ is the combustion temperature, 573 K.

After calculation, we can get the exergy efficiency of the gas boiler system *ε*_*g*Π_ being 43.9%.

### 3.3. Annual Heating Cost Analysis of RWSHPS

The output exergy costs [11] of energy conversion system are composed of the cost of energy and nonenergy costs. The heat exergy and electricity exergy are energy costs, but the annual cost of the system is nonenergy cost. The total cost of the system is the sum of energy cost and nonenergy costs:

(10) $$\begin{array}{c} C_{\text{pr}}=C_{\text{En}}+Z_{a}. \end{array}$$

Thermoeconomics cost includes not only the external energy exergy cost but also the cost of investment and operation management costs in the process of production; it reflects the economic efficiency of the process of the production.

#### 3.3.1. Energy Cost of the RWSHPS

System operating costs mainly include the electricity cost of each part; water charges within the secondary network are not considered here. There are several known conditions on the energy cost calculation.

1. In Shijiazhuang, one heating season contains 120 days, from 15 November to 15 March, According to running experience, in the period of 15 November to 15 December and 15 February to 15 March, the loading rate of the system is 0.5, and in the last two months of the heating period, one is at full capacity, and the loading rate of the rest of the month is 0.8.
2. The project belongs to the livelihood projects; the Reclaimed water entering into the system is free of charge; namely, the cost of logistics exergy *c*_0_ entered into the system is equal to 0.
3. The electricity price is 0.52 yuan/(kWh), calculated in accordance with the public electricity prices; the data are listed in Table 1.

#### 3.3.2. Nonenergy Cost of the RWSHPS

Annualized cost includes the investment costs and management costs of the system. We need to consider the time value of the capital, such as the salvage value of the system and the annual operation and management fees. The calculation of the nonenergy costs follows some known conditions.

(1) The lifetime of the equipment in the system is 20 years; the salvage value of the heat pump units, pumps, and other equipment (SV1) accounts for 10% of the initial investment in the system; and the salvage value of the transmission and distribution network (SV2) accounts for 50% of its initial investment.
(2) Annual interest rate *i* is equal to 5%; the cash coefficient PWF (*i*, *n*) and the capital recovery coefficient CRF (*i*, *n*) are
  (11) $$\begin{array}{c} \text{PWF}\left(i,n\right)={\left(1+i\right)}^{-n}=1.05^{-20}=0.38,\\ \text{CRF}\left(i,n\right)=\frac{i}{1-{\left(1+i\right)}^{-n}}=\frac{0.05}{1-0.38}=0.08. \end{array}$$

The annualized cost *Z*_*i*_ is calculated by

(12) Zi={[C0−Sv×PWF(i,n)]  +∑mnFm×PWF(i,n)}×CRF(i,n),

where *C*_0_ is the initial investment,  yuan; *F*_*m*_ is the operation and management fees in the year of *m*, yuan. The nonenergy cost calculations of the RWSHPS are shown in Table 2.

### 3.4. Heating Costs of the Gas Boiler System

#### 3.4.1. The Energy Cost of the Gas Boiler System

 The gas boiler heating system mentioned before is designed for a residential quarters of 300,000 m^2^; its energy consumption is listed in Table 3.

#### 3.4.2. Nonenergy Cost of the Gas Boiler System

The calculation of the nonenergy costs of the gas boiler heating system is the same as the RWSHPS, which also includes annualized cost of the initial investment and the annualized cost of the operation and management fees in its lifetime:

(13) Zg=λgX+λgY,Pg=[Cg0−Sgv×PWF(i,n)] +∑mnFgm×PWF(i,n),Zg1=Pg×CRF(i,n).

The initial investment of the entire gas boiler heating system is

(14) $$\begin{array}{c} C_{g0}=C_{g1}+C_{g2}+C_{g3}, \end{array}$$

where *C*_*g*0_ is the total cost of the gas boiler plant (10^4^  yuan) and

*C*_*g*1_ is the equipment costs of gas boiler plant [12]:

(15) $$\begin{array}{c} C_{g1}=16.8Q_{1}=16.8\times 12.4=208\;\left(10^{4}\text{ yuan}\right). \end{array}$$

*C*_*g*2_ is the based on the actual experience; gas boiler room installation costs accounted for 70% of the equipment costs:

(16) $$\begin{array}{c} C_{g2}=0.7C_{g1}=0.7\times 208=145.6\;\left(10^{4}\text{ yuan}\right). \end{array}$$

*C*_*g*3_ is the civil engineering costs of the gas boiler room:

(17) $$\begin{array}{c} C_{g3}=0.13A=0.13\times 355=46.2\;\left(10^{4}\text{ yuan}\right). \end{array}$$

After calculation, we can get the total cost of the gas boiler plant:

(18) Cg0=208+145.6+46.2=400 (104  yuan),Pg=[400−40×0.38]+20×54.24×0.38=797 (104  yuan),Zg1=Pg×0.08=797×0.08=64 (104  yuan).

The heating costs of gas boiler plant in a heating season are

(19) $$\begin{array}{c} C_{g\text{pr}}=C_{g\text{En}}+Z_{g1}=737+64=801\;\left(10^{4}\text{ yuan}\right). \end{array}$$

Unit heating cost is 26.7 yuan.

### 3.5. The Comparison of the Two Heating Methods

Through the analysis and calculation above, some parameters of the two different heating methods are listed in Table 4.

## 4. Conclusions

We take the exergy analysis method and the thermoeconomic cost analysis method as the theoretical basis of this paper, using the method of theoretical analysis to qualitatively describe that it is reasonable for us to use heat pump systems for district heating, giving the calculation method of energy, efficiency and exergy efficiency. Analysis pointed out that only taking into account these two methods can get a more comprehensive evaluation on energy conversion system. The aspects of heating costs, energy, and exergy efficiency, and so forth were compared. Through research, this paper obtained the following conclusions.

(1) This work describes a large Reclaimed water source heat pump system (RWSHP) and elaborates on the composition of the system and its design principles, designing a Reclaimed water source heat pump system which will match the heating needs of 8 million square meters. According to the characteristics of the Reclaimed water and save the initial investment, the project is divided into two stages: the first stage adopts distributed heat pump heating system and the second adopts the combination of centralized and decentralized systems.

(2) After thermodynamic analysis of the two heating methods, we can see that the exergy efficiency of the RWSHPS is relatively high, being, respectively, 66.2% and 60.6% of the project phase I and II, while gas boiler system is 43.9%. In terms of energy consumption costs, the energy costs of the gas boiler system are twice as much as RWSHPS. On the one hand, there is a relatively large exergy loss in the process of gas combustion in the gas boiler, and the temperature of the smoke leaving system is up to 180°C. On the other hand, it takes away a lot of useful energy, resulting in a larger amount of energy loss due to a higher unit price of natural gas. On the contrary, the Reclaimed water is the heat source of the RWSHPS, which belongs to the waste recovery and reuse, so the running costs of the RWSHPS are relatively low.

(3) By the analysis of the two systems using the cost theory of thermoeconomics, it is shown that the initial investment of the RWSHPS is more than three times of that of the gas boiler plant system. Because of that the RWSHPS includes the construction costs of transmission and distribution network. This part of investment can be offset by charging the pipeline network construction fees to users. The heating cost of project phase I of the RWSHPS is 16.3 (yuan/m^2^·heating season); the heating cost of project phase II is 17.2 (yuan/m^2^·heating season), while that of the gas boiler system is 26.7 (yuan/m^2^·heating season). The unit heating cost of the RWSHPS is less than that of the gas boiler heating system by about 10 yuan. So it can be seen that the RWSHPS can be implemented widely. There is a greater potential for profit. This paper also provides a reference for future run enterprises.

## Figures and Tables

**Table 1:** Energy costs of RWSHPS.

Engineering phase	Electrical load (kW)	Power consumption (kWh/a heating season)	Energy cost in a heating season (ten thousand yuan)	Unit energy costs (yuan/m^2^·a heating season)
Project phase I	20340	42 430 752	2206	10.9
Project phase II	100718	203 047 488	10559	13.2

**Table 2:** Nonenergy costs of RWSHPS.

	Investment cost			Management costs	Annualized cost
Engineering phase	Heat pump room (ten thousand yuan)	pipeline network (ten thousand yuan)	Circulation pump station (ten thousand yuan)	(Ten thousand yuan)	(Ten thousand yuan)
Project phase I	5526	5278	540	186	1082
Project phase II	31227	5278		805	3235

**Table 3:** The energy cost of the gas boiler system.

	Quantity	Unit price (yuan)	Total (ten thousand yuan)	Total energy costs (ten thousand yuan/a heating season)	Unit energy costs (yuan/m^2^·a heating season)
Fuel consumption	2937312 (m^3^/a heating season)	2.4	705	737	24.6
Auxiliary power consumption	324576 (kW/a heating season)	0.8	26		
Water consumption	24192 (m^3^/a heating season)	2.4	5.8		

**Table 4:** Comparison of the two heating methods.

Heating scheme	Heating area (10^4^ m^2^)	Initial investment cost (10^4^ yuan)	heating cost (yuan/m^2^·a heating season)	Unit energy costs (yuan/m^2^·a heating season)	Exergy efficiency (%)
RWSHPS	200	11344	16.3	10.9	66.2
	800	36505	17.2	13.2	60.6
Gas boiler system	800	10800	26.7	24.6	43.9
